# A Novel Cause of CIDP: Homozygous Hotspot Mutation, c.793 C > T in CASP8 Gene

**DOI:** 10.1007/s10875-025-01931-w

**Published:** 2025-09-30

**Authors:** Kamal Sharma, Ana Flores, Paul Maertens

**Affiliations:** 1 University of South Alabama, Mobile, USA; 2 Department of Pediatrics, Pediatric Critical Care Division, University of South Alabama, 1700 Center Street, 36604 Mobile, USA

**Keywords:** Genetic cause, Areflexia, Polyneuropathy

## Abstract

**Background:**

Caspase-8 enzyme deficiency (CED) is a rare autosomal recessive inborn error of immunity with autoimmune lymphoproliferative syndromes (ALPS), deficient extrinsic apoptosis and hyperactivation of the mammalian target of rapamycin (mTOR) pathway.

**Methods:**

Features of our patient with early onset chronic inflammatory demyelinating polyneuropathy (CIDP) are presented in detail. We report features of ALPS due to CED (ALPS-CED) in 6 patients in previous literature and our patient with the homozygous hotspot mutation, c.793 C > T in the CASP8 gene, and demonstrate that such mutation is a novel cause of CIDP.

**Results:**

Our patient fits the spectrum of genetic disorders causing ALPS with dysregulation of the mTOR pathway. Autoimmune lymphoproliferative syndrome with FAS mutations (ALPS-FAS) is also associated with hyperactivation of the mTOR pathway but hematologic symptoms are more severe than ALPS-CED. Both ALPS-FAS and ALPS-CED lead to autoimmunity and improve with use of mTOR inhibitors. ALPS-FAS is characterized by the elevation of alpha beta-double negative T-cells (DNT) and hypergammaglobulinemia, while such features are not prominent in ALPS-CED. Patients with ALPS-CED not only suffer from ALPS but also present with an immune deficiency and a chronic inflammatory state both related to non-apoptotic functions of caspase-8.

**Conclusions:**

Both ALPS-FAS and ALPS-CED are characterized by deficient extrinsic apoptosis and dysregulation of the mTOR pathway. Compared to ALPS-FAS, the phenotype of ALPS-CED is more complex due to a more diffuse dysfunction of the non-apoptotic pathways. To our knowledge, early onset CIDP in a patient with homozygous hotspot mutation, c.793 C > T in CASP8, has not been reported previously.

## Background

Most (~ 85%) polyneuropathies occurring in children are hereditary [1]. Guillain-Barré syndrome (GBS) and chronic inflammatory demyelinating polyneuropathy (CIDP) are autoimmune conditions resulting from T cell-mediated and humoral immunity against antigens of myelin in Schwann cells and/or axonal neurofilaments. Autoimmunity as seen in GBS and CIDP is responsible for ~ 9% of childhood polyneuropathies. CIDP is uncommon compared to GBS, and its prevalence rate is 0.22 children per 100,000 [2]. Onset as early as infancy is well documented in patients with CIDP [3]. Presenting symptoms of GBS and CIDP are similar and include symmetric worsening of muscle weakness, sensory dysfunction, and decreased or absent deep tendon reflexes. Clinically, temporal evolution differentiates CIDP from GBS. In GBS, symptoms progress rapidly over days to weeks. GBS is a monophasic, self-limited autoimmune illness leading to recovery and occurring only once. Neurologic deficits in CIDP are more insidious and usually worsen gradually over more than 8 weeks. In CIDP, the autoimmune response is persistent, leading to a relapsing-remitting or a slowly progressive course.

In both GBS and CIDP, there is failure of immunological self-tolerance and homeostasis. In GBS, the percentage of circulating memory B cells gradually decreases during the recovery phase [4]. In the peripheral nervous system, activated T-cells that crossed the blood brain barrier are eliminated by apoptosis [5]. In GBS, lymphocytic apoptosis is of crucial importance in eliminating lymphocytes with antigen receptors that recognize autoantigens, thereby preventing persistent autoimmunity. In CIDP, the percentage of memory B cells remains elevated even with remission and stable disease status [6]. In the peripheral nervous system, defective apoptosis of the activated T-cells is one of the mechanisms contributing to the chronic course of CIDP [5]. We demonstrate that our patient with CIDP has phenotypic characteristics of the autoimmune lymphoproliferative syndrome (ALPS) due to caspase-8 enzyme deficiency (ALPS-CED). The autoimmune dysregulation is associated with atopic dermatitis, recurrent infections, and failure to thrive.

## Case Presentation

We present a 6-yr-old male born at 37 weeks of gestation by vaginal delivery with birth weight of 3487 gm. He was breastfed until 2 years of age. At 2 months of age, he presented with a recurrent pruritic rash with dry patches on the antecubital fossa, popliteal fossa, abdomen, and buttocks, which flared up every two weeks. He was diagnosed with atopic dermatitis and treated with topical steroid cream. Sleep was poor. He had intermittent fever of unknown etiology and upper respiratory symptoms. He never had gastroenteritis. Recurrent ear infections started at 3 months of age requiring a first myringotomy at 9 months and a second myringotomy at 12 months. Neurologic development was normal until 6 months of age when he sat up without Support. However, by 10 months of age, he was still not crawling. Weight gain was poor. After a period of developmental stagnation, developmental regression with increasing weakness started at 13 months. Speech and language were relatively spared. By 15 months, he was severely wasted and unable to sit. 

At 19 months of age, he presented to our institution with respiratory distress and was bedridden. Weight was only 7800 gm (below 2nd percentile). Breath sounds were coarse with mild retraction, and secretion clearing was impaired. Severe muscle wasting was accompanied by generalized muscle weakness, hypotonia, and areflexia. Cranial nerves II to XII were intact. He was fully aware and responsive, moving all four extremities to commands. Skin rash was noted in the perineal region. He also suffered from molluscum contagiosum. Large cervical, axillary, and inguinal adenopathies were noted. 

Workup included normal creatinine kinase and normal brain MRI. Spinal MRI with gadolinium showed diffuse enhancement of the roots and cauda equina. CSF showed 7 white cells (87% lymphocytes, 12% monocytes), no red cells, glucose of 63 mg/dL, protein of 140 mg/dL (N 15–45), albumin (79 mg/dL; N < 26) and IgG (12 mg/dL; N < 6). IgG synthesis was high (16.1 mg/day). There were no oligoclonal bands on immunofixation. Erythrocyte sedimentation rate was normal (15 mm/hour). Serum antinuclear antibodies were weakly positive (1.9 U; N is less than 1.0 U) while serum cyclic citrullinated peptide antibody was normal (16.7 U; N < 29 U). Plasma IgA (35 mg/dL; N: 9–107), IgG (925 mg/dL; N: 700–1600) and IgM were normal (48 mg/dL; N: 35–239). The patient was diagnosed with acute on chronic respiratory failure and treated with antibiotics and oxygen support.

At 18 months of age, nerve conduction study showed prolonged distal onset motor latency (DML) (L median: 12.8 ms; L ulnar: 13.6 ms) and absent F-wave (FW) potential with slow nerve conduction velocity (NCV) in the demyelinating range, associated with decreased compound motor action potential (CMAP) amplitude and temporal dispersion in the left median (L: 0.4 mV) and ulnar motor nerves (L: 1.0 mV). Left median and ulnar sensory nerves showed no response. Prolonged DML (L peroneal: 10 ms; R peroneal: 18.8 ms; L post tibial: 9.6 ms; R post tibial: 13.6 ms) in the demyelinating range, no FW potentials and slow NCV in the demyelinating range were associated with temporal dispersion and decreasing CMAP in bilateral peroneal motor and posterior tibial motor nerves. Bilateral sural sensory nerves showed no response. Bilateral peroneal sensory nerves were not evaluated.

Muscle biopsy showed neurogenic atrophy and sural nerve biopsy showed inflammatory features with endoneurial mononuclear cells and demyelination with onion bulb formation consistent with chronic inflammatory demyelinating polyneuropathy. Immunohistochemical studies showed frequent scattered individual endoneurial cells, multiple endoneurial perivascular, and occasional epineurial perivascular collections of cells with CD-45 (leukocyte common antigen) staining. Cells showed reactivity to a macrophage (CD-68) preparation. There was a small endoneurial perivascular collection of inflammatory cells which was pan-positive for CD-3, CD-4, CD-8, and CD-20. An additional small endoneurial perivascular collection was present which was predominantly CD-3 positive with CD-8 positivity as well but essentially no staining for CD-4 or CD-20. Immunohistochemical staining of individual cells scattered throughout the endoneurium was strongly positive for CD-3, less positive for CD-8, and essentially negative for CD-4 or CD-20.

In our patient, a blood sample was sent for a comprehensive hereditary neuropathy panel, performing full-gene sequencing and deletion/duplication analysis using next-generation sequencing technology. No mutation was found in genes that are associated with hereditary neuropathies, including but not limited to Charcot-Marie-Tooth disease (CMT), hereditary motor neuropathy (HMN), and hereditary sensory and autonomic neuropathy (HSAN).

Neurologic status improved slowly with IVIG every two weeks and weekly steroids. At 3 years of age, rituximab 375 mg/m^2^ without alteration of IVIG and steroid regimen was followed by a progressive slow improvement. He finally became ambulatory at 5 years of age. He had severe kyphoscoliosis which required spinal fusion and instrumentation at 10 years of age. Foot deformities became severe interfering with ambulation. He graduated from high school with honors at the age of 18. Whole exome sequence (WES) analysis showed that our patient is homozygous for an autosomal recessive known pathogenic CASP8 variant (NM_001228.4: c.793 C > T, p.R265W). The parents were both carriers of the same mutation, and although not consanguineous, were distantly related as great grandfathers of both parents (four generations back) were brothers. Flow cytometry on whole blood in our patient demonstrated normal double negative T cell receptors (CD3^+^TCRαβ^+^CD4^–^CD8– T cells) as % alpha/beta-TCR DNT was 1.8% (N < 2%). Quantification of lymphocyte subsets showed normal CD4 T cells (41%; N 31–52), high CD8 T cells (44%; N 18–35), low CD19 B cells (5%: N: 6–23) and normal CD16 + CD56 NK cells (8%; N: 3–22). CD4/CD8 ratio was low (0.92; N > 1). Plasma levels of B12 were not elevated (398; N: 180–914 ng/L). Plasma cytokine panel showed elevated tumor necrosis factor-alpha (TNF-α) (16.1; N < 10 pg/mL) and a slight elevation of IL-18 (816; N < 468 pg/mL) and IL-10 (7.7; N < 7pg/mL).

Motor and sensory nerve conduction studies were repeated at 21 years of age. There was only an evaluation of the upper extremities. The left and right median motor nerves showed prolonged DML (L 16.4, R 33.3 ms), no response at the elbow and reduced CMAP (L 0.4, R 0.4 mV). The left ulnar motor and the right ulnar motor nerves showed prolonged DML (L 8.4, R 7.0 ms), reduced amplitude (L 0.7, R 0.4 mV), and decreased conduction velocities (below elbow (BE)-wrist (W): L 11, R 14 m/s) (above elbow (AE) to BE: L 15, R 23 m/s). The bilateral median sensory, right radial sensory and bilateral ulnar sensory nerves showed no response (wrist). The left radial sensory nerve conduction was within normal limits. FW studies indicated that the left median FW had no response, the right median FW had prolonged latency (41.08 ms) and the left ulnar FW had no response.

## Results & Discussion

WES analysis reveals that our patient is homozygous for a previously described autosomal recessive missense pathogenic variant in exon 8 of the CASP8 gene at chromosome position chr2:g.202,141,631 C > T. The transcript found is c.793 C > T, p.R265W (NM_001228.4). Both parents are heterozygous for the p.R265W variant in the CASP8 gene. Each laboratory selects its own reference transcript, and as a result, there is great variability in the transcripts utilized by sequencing laboratories [7]. The same mutation has alternate notations based on other transcripts (NM_033355 c.742 C > T, p.R248W, NM_001080125 c.919 C > T, p.R307W and NM_001080124 c.697, C > T, p.R233W). The inheritance mode of pathogenic missense mutations causing caspase-8 enzyme deficiency (CED) is autosomal recessive. Pathogenic homozygous mutations of CASP8 result in loss of function and as expected, are mainly observed in the buried region of the protein, causing destabilization of the protein structure [8]. This missense variant replaces an arginine residue at position 265 with tryptophan. Arginine is a highly hydrophilic basic amino acid while tryptophan is a highly hydrophobic neutral, non-polar aromatic amino acid. This variant disrupts the protein’s secondary and tertiary structures by causing the protein to fold away from the newly introduced hydrophobic region. This change is in a conserved amino acid in the p18 domain of caspase-8, affecting the catalytic domain of the protein. In silico analysis Supports that this missense variant has a deleterious effect on the protein structure and function. In large population cohorts, this specific allele variation is observed in 0.0014% of individuals (4 out of 282930 alleles), and no individuals are reported as homozygous [9]. Published functional studies demonstrate a damaging effect of the mutation [10]. 

The homozygous missense c.793 C > T mutation in the CASP8 gene is a hotspot mutation that has been reported in six patients in the previous Literature. Patients 1 and 2 were siblings of consanguineous parents [10]. Their neurologic development was normal, but they Suffered from lymphoproliferative combined immunodeficiency as both had lymphadenopathies, mild splenomegaly, early onset sinopulmonary bacterial infections, and severe mucocutaneous herpes infection. Both children had skin rash and failure to thrive. Patient 2 also suffered from chronic diarrhea [10]. Patient 3 and patient 4, who are siblings, were born to parents distantly related to patient 1 and patient 2 [11]. In both, the onset of their symptoms was in adulthood, in their late thirties. Patient 3 presented with shortness of breath. A lung biopsy showed multiple necrotizing and non-necrotizing granulomas. Nodular lymphocytic hepatosplenomegaly was an additional feature. Patient 4 presented with a neurological disease leading to multiple cranial nerve palsies. A left Meckel’s cave necrotizing granuloma was found [11]. Patient 5 presented in the first year of life with failure to thrive and chronic diarrhea due to very early onset irritable bowel disease and refractory proctocolitis [12]. There was an increased susceptibility to bacterial and viral infections [12]. Patient 6 was first evaluated for rash and fever at 5 months of age [13]. He Suffered from recurrent fever, failure to thrive, sinopulmonary infection, chronic diarrhea, persistent generalized lymphadenopathies first noticed at 1 year of age, and hepatosplenomegaly [13]. To our knowledge, we are the first to report infantile onset CIDP in a patient with CED. Our patient presented in the first year of life with recurrent fever of unknown etiology, recurrent otitis media, failure to thrive, and eczema/atopic dermatitis. Persistent generalized lymphadenopathy was first noted in the second year of Life. He was noted to have a progressive motor delay after 6 months of age. Our patient never had diarrhea but was still breastfed when diagnosis of CIDP was first Suspected at 19 months of age due to hyporeflexia.

CED is an inborn error of immunity characterized by an ALPS, a deficient extrinsic apoptosis, and hyperactivation of the mTOR pathway [14]. ALPS is typically caused by mutations in the tumor necrosis factor receptor Superfamily 6 gene which regulates extrinsic apoptosis and encodes a transmembrane protein (Fas) with intracellular death domain (DD). The ALPS-FAS phenotype is also reported in Fas ligand (FasL) gene mutations [15]. The primary function of the Fas/FasL system is the induction of extrinsic apoptosis in activated T and B lymphocytes and NK cells and macrophages that are no longer needed, thereby preventing excessive immune response and maintaining immune tolerance. This eliminates potentially harmful or autoreactive immune cells and maintains immune system homeostasis [16]. When Fas is activated by its ligand, the intracellular death domain of Fas recruits an adaptor protein called FADD (Fas-associated death domain protein), which then binds to and recruits procaspase-8.

Cytoplasmic caspase-8 recruited by the intracellular death domain of Fas is the primary initiator protease of the extrinsic apoptosis. Non-apoptotic functions of cytoplasmic caspase-8 (in non-apoptotic cells) include the cleavage of cytoskeletal proteins like focal adhesion components, impacting cell dynamics and behavior [17]. A small amount of caspase-8 is also found in the nucleus of non-apoptotic cells. The most important non-apoptotic function of nuclear caspase-8 is cleavage and inactivation of specific deubiquitinases, preventing them from deubiquitinating and stabilizing target proteins, thereby affecting protein stability and downstream signaling pathways. In non-apoptotic cells, caspase-8 prevents the deubiquitinase, ubiquitin-specific peptidase 7 (USP7), from deubiquitinating PTEN (phosphatase and tensin homolog). Monoubiquitinated PTEN remains in the nucleus where it protects chromosomes by directly interacting with centromeric proteins and by associating with histone and other chromatin proteins, thereby maintaining proper chromatin structure and stability [18]. 

When Fas is activated, caspase-8 is translocated from the nucleus to the plasma membrane, releasing USP7 from caspase-8 inactivation [19]. During apoptosis, USP7 is released from non-apoptotic caspase-8 inhibition, deubiquitinating PTEN. Deubiquitinated PTEN translocates out of the nucleus and functions at the cell membrane level as a lipid phosphatase, inhibiting the PI3K/AKT/mTOR signaling pathway by dephosphorylating phosphatidylinositol trisphosphate (PIP3) [20]. 

Clinically ALPS-CED resembles ALPS-FAS (Table 1) [21]. ALPS-FAS is a chronic (> 6 months) noninfectious and nonmalignant lymphoproliferative disease characterized by lymphadenopathies in at least two sites and/or often massive splenomegaly [21]. Hepatomegaly is less frequent. Clinical manifestations of lymphoproliferation develop in childhood usually before age 5 years [22]. Five previously published patients and our patient with the same homozygous mutations responsible for ALPS-CED had lymphadenopathy. In our patient, lymphadenopathies were more prominent in early childhood. Splenomegaly was present but mild in four ALPS-CED patients. Hypergammaglobulinemia is a common feature of ALPS-FAS, but paradoxically, fewer conventional memory cells differentiate from FAS-expressing germinal center B cells, while extrafollicular splenic B cells are enhanced [23]. ALPS-FAS with hyperactivation of the mTOR pathway, is characterized by the accumulation of double-negative T cells (DNTs), and a population of T cells lacking both CD4 and CD8 markers, often exceeding 2.5% of the total T cell population. Inhibition of the DNT cells in ALPS-FAS is unique since they produce high IL-10 and display surface markers such as CD27 and CD28, which have the characteristics of long-lived memory T cells with potent proliferative capacity [14]. The clinical presentation of ALPS-FAS is also associated with high levels of vitamin B12, interleukin IL-10, IL-18, and soluble FASL, and impaired FAS-mediated apoptosis [24]. Most patients with ALPS-CED with the same mutation as our patient were never investigated for biomarkers of ALPS. In contrast to patients with ALPS-FAS, ALPS-CED is not associated with hypergammaglobulinemia. Hypogammaglobulinemia is reported in some patients. DNTs have been normal or borderline high in the ALPS-CED siblings reported by Chun [10]. DNT cells and B12 are not elevated in our patient. IL-10 and IL-18 are high in our index patient (Table 2). High levels of IL-10 indicate an abnormal immune response where the body is unable to properly eliminate damaged or unnecessary cells due to impaired apoptosis, leading to potential autoimmune complications; essentially, the elevated IL-10 acts as an immunosuppressive signal, hindering the immune system’s ability to clear abnormal cells.

**Table 1:** Clinical features of ALPS

Clinical feature	^a^ALPS-FAS	ALPS-CED patients						
		^b^Pt1	^b^Pt2	^c^Pt3	^c^Pt4	^d^Pt5	^e^Pt6	Our Pt
Age of onset (yr)	Childhood	—	—	37	38	< 1	< 1	< 1
Failure to Thrive	Occasional	Yes	Yes	No	No	Yes	Yes	Yes
Lymphadenopathy	Yes	Yes	Yes	—	Yes	—	Yes	Yes
Splenomegaly	Yes	Mild	Mild	—	Yes	—	Yes	No
Neurologic symptom	Occasional	Bell’s palsy	Bell’s palsy	Trigeminal neuralgia, cranial neuropathy	—	—	No	CIDP
Hematologic autoimmunity	Yes	Yes	Yes	—	—	—	No	No
Coombs positive AB	Yes	Yes	Yes	—	—	—	—	—
Systemic autoantibody	Yes	Yes	—	—	—	—	—	Yes
Lymphoma	Yes	—	—	—	—	—	—	—
Recurrent infection	No	Yes	Yes	Yes	Yes	—	Yes	Yes
Rash	Rare	Yes	Yes	—	—	—	Yes	Yes
IBS	No	No	Yes	No	No	Yes	Yes	No

Abbreviations: *ALPS* autoimmune lymphoproliferative syndrome, *ALPS-FAS* ALPS due to Fas mutation, *ALPS-CED* ALPS due to Caspase 8 deficiency, *CIDP* chronic inflammatory demyelinating polyneuropathy

^a^ Hafezi N et al., ^b^ Chun HJ et al., ^c^Niemela J et al., ^d^ Lehle AS et al., ^e^ Chougule AS et al. are referenced in the manuscript

**Table 2:** Laboratory findings in ALPS-FAS and ALPS-CED patients

	^a^ALPS-FAS Patients	ALPS-CED patients							Range
		^b^Pt1	^b^Pt2	^c^Pt3	^c^Pt4	^d^Pt5	^e^Pt6	Pt7	
DNTs (%)	High	3.3	4.6	1.7	—	—	0.8	1.8	< 2
Vitamin B12 (ng/L)	High	—	—	—	—	—	—	398	180–914
IL10 (pg/ml)	High	—	—	—	—	—	—	7.7	< 7
IL18 (pg/ml)	High	—	—	—	—	—	—	816	< 468
IgG (mg/dl)	High	857	544	1030	1124	—	1700	925	760–1550
IgA (mg/dl)	High	112	56	181	117	—	181 (H)	96	70–400
IgM (mg/dl)	N or Low	57	32	22	15	—	126	48	35–239
IgE (mg/dl)	High	7	20	46	17	—	—	—	< 90
Lymphocyte/µl	N or High	1510	2362	3719	990	—	13,505	1550	2300–5400
CD4 (%)	N	23.	25	9.3	23.5	—	18	41	25–48
CD8 (%)	N or High	50	46.2	87.6	65.5	—	57	44	9–35
CD4/CD8 ratio	Low	0.5	0.5	0.1	0.35	—	0.32	0.92(L)	0.9–3.4
B Lymph (%)	N or High	23.9	24.1	—	—	—	10	6.3	8–23
NK cells (%)	Low or N	—	—	—	—	—	8.99	12.4	6–27

Abbreviations: *ALPS* autoimmune lymphoproliferative syndrome, *CIDP* chronic inflammatory demyelinating polyneuropathy, *N* normal

^a^ Hafezi N et al., ^b^ Chun HJ et al., ^c^Niemela J et al., ^d^ Lehle AS et al., ^e^ Chougule AS et al. are referenced in the manuscript

Autoimmunity is a feature of ALPS-FAS and ALPS-CED. Many patients with ALPS-FAS have a variable combination (2 or more) of autoimmune cytopenia such as autoimmune hemolytic anemia, neutropenia, and thrombocytopenia [25]. In one study, it was shown that autoantibodies to neutrophils, red blood cells, and platelets are important markers of ALPS, frequently detected before autoimmune disease manifests clinically [26]. Cytopenia is not a prominent feature of ALPS-CED. The siblings reported by Chun exhibited direct Coombs positive antibodies with a compensated mild hemolytic anemia [10]. Cytopenia was never a feature in our patient. Non-hematologic autoimmune features of ALPS-FAS are uncommon but possible [27] and include autoimmune hepatitis, pulmonary fibrosis [28], GBS [29], or transverse myelitis [30]. Smooth muscle antibodies, anticardiolipin autoantibodies, antinuclear autoantibodies, and rheumatoid factors can be seen in some ALPS-FAS patients, even if asymptomatic [26]. Non hematologic autoimmune features of ALPS-CED are more severe. In one patient, the lung is affected causing adult onset autoimmune-mediated pulmonary fibrosis. In another patient, the peripheral nervous system was affected by adult-onset multiple cranial nerve neuropathy [11]. Infantile onset CIDP was seen in our patient. Anti-thyroid antibodies without thyroid disease are reported in the CED siblings reported by Chun [10]. One CED patient has lupus anticoagulant with prolonged PTT but no history of thrombotic disease [10]. Our patient was weakly positive for antinuclear antibodies.

Primary immune deficiency is a rare feature of ALPS-FAS. An infant with homozygous FasL mutation had recurrent otitis media in infancy [31]. ALPS-FAS does not significantly raise the risk for frequent or severe infections. However, ALPS-FAS patients with autoimmune neutropenia or post therapeutic splenectomy have an increased susceptibility to infection [32]. Increased susceptibility to infection is a feature of ALPS-CED. All individuals with ALPS-CED due to the same homozygous mutation as our patient had increased risk for viral and bacterial infections. Immunodeficiency due to CED is characterized by recurrent sinopulmonary infections, herpes simplex virus infections, and poor responses to immunization [10]. Our patient had early onset and frequent otitis media requiring tympanostomy tube placement despite the protective effect of prolonged breastfeeding [33]. B-cell immunodeficiency is not a feature of ALPS-FAS [34], or ALPS-CED. B cells are low in our patient, most likely due to the chronic use of IVIG [35]. An inverted CD4/CD8 ratio is not seen in ALPS-FAS but is a feature of ALPS-CED. When Toll-like receptors (TLRs) on T, B, or NK cells are activated by a pathogen-associated molecular pattern, it can trigger a signaling cascade that leads to the activation of nuclear caspase-8, ultimately contributing to the T cell response by promoting cytokine production and cell proliferation [36, 37]. Such function of caspase-8 is non-apoptotic. CD4 helper T cells lead the fight against infections and when they become activated, they coordinate the immune response by releasing cytokines that signal other immune cells to act. CD8 cytotoxic T cells recognize and produce molecules that directly kill infected host cells. A lack of CD4 cells leads to more frequent infections. A CD4:CD8 ratio of less than 1 indicates an impaired immune system [38]. 

Inflammation is a rare feature in ALPS-FAS. A cutaneous rash was reported in one child with homozygous FAS mutation and ALPS-FAS [39]. A transient skin rash was also reported shortly after birth in an ALPS-FAS patient with homozygous mutation [40]. Chronic diarrhea can be a feature of ALPS-FAS. It has also been reported in one infant with homozygous FASL mutation [41]. Atopic dermatitis is a common feature of ALPS-CED. Four of the patients with the same CASP8 mutation, including ours, had atopic dermatitis. In mice, CED promotes the pathogenesis of atopic dermatitis [42]. Very early onset inflammatory bowel disease (VEOIBD) is a feature of ALPS-CED. It is present in three patients with the same mutation but not in our patient. Caspase-8, in response to stimuli Like tissue damage or infection, cleaves the cytoplasmic receptor-interacting protein kinase 1 and 3 (RIPK1-RIPK3), preventing the formation of a complex that would otherwise trigger necroptosis, a highly inflammatory form of cell death. In ALPS-CED, this non-apoptotic function of caspase-8 is deficient. The inflammation seen in atopic dermatitis and VEOIB is not primarily caused by a lack of apoptosis but rather by dysregulation of cytoplasmic RIPK1-RIPK3 signaling, which proceeds unchecked, leading to necroptosis [43, 44]. 

In addition to the systemic features of ALPS-CED described above, our index patient presented with a rare infantile onset CIDP, an autoimmune disorder where the myelin of peripheral nerves and nerve roots is damaged. CIDP is a syndrome based on clinical, electrophysiologic, and pathologic criteria [45]. Our patient fulfilled clinical criteria as symptoms developed progressively over more than 8 weeks. Symptoms included distal and proximal weakness, sensory loss and diminished or absent deep tendon reflex in all four extremities. Electrophysiologic studies showed diffuse motor and sensory nerve conduction findings compatible with demyelination and axonal damage. Pathologically, sural nerve biopsy revealed onion bulb formation, the hallmark of recurrent demyelination and remyelination. There was chronic scattered and perivascular endoneurial inflammation with increased total T cell number.

Pathogenic mechanisms of CIDP remain elusive as the contribution of genetic and environmental factors can vary across various groups of patients. A more pronounced expansion of CD8 T cells compared to CD4 T cells was seen in our patient. CD8 T cells (often called cytotoxic T lymphocytes) express Fas and procaspase 8 on their surface. In GBS, Fas-mediated T cell apoptosis is the mechanism by which CD8 T cells commit fratricide, eliminating each other during the contraction phase at the end of an immune response. Defective Fas-mediated T cell apoptosis is seen in CIDP, suggesting that the inability to kill CD8 T cells plays a role in the chronic evolution of CIDP [46]. When Fas-mediated apoptosis is deficient, as in our patient with CED, the inflammation is caused by dysregulation of cytoplasmic RIPK1-RIPK3 signaling, which proceeds unchecked leading to necroptosis. More research is needed to fully understand the interplay between RIPK1, RIPK3, and both pro- and anti-inflammatory cytokines in CIDP.

## Conclusions

To our knowledge, early onset CIDP in a patient with homozygous hotspot mutation, c.793 C > T in the CASP8 gene, has not been previously reported. Both ALPS-FAS and ALPS-CEDS are characterized by dysregulation of the mammalian target of rapamycin (mTOR) pathway. Compared to ALPS-FAS, the phenotype of ALPS-CED is more complex as indicated due to a more diffuse dysfunction of the non-apoptotic pathways.

## Data Availability

No datasets were generated or analysed during the current study.
